# Lampreys and spinal cord regeneration: “a very special claim on the interest of zoologists,” 1830s-present

**DOI:** 10.3389/fcell.2023.1113961

**Published:** 2023-05-09

**Authors:** Kathryn Maxson Jones, Jennifer R. Morgan

**Affiliations:** ^1^ Center for Medical Ethics and Health Policy, Baylor College of Medicine, Houston, TX, United States; ^2^ Department of History, Purdue University, West Lafayette, IN, United States; ^3^ Marine Biological Laboratory, The Eugene Bell Center for Regenerative Biology and Tissue Engineering, Woods Hole, MA, United States

**Keywords:** lampreys, Petromyzon marinus, regenerative medicine, neuroscience, aquatic biology, model organisms, reticulospinal neurons

## Abstract

Employing history of science methods, including analyses of the scientific literature, archival documents, and interviews with scientists, this paper presents a history of lampreys in neurobiology from the 1830s to the present. We emphasize the lamprey’s roles in helping to elucidate spinal cord regeneration mechanisms. Two attributes have long perpetuated studies of lampreys in neurobiology. First, they possess large neurons, including multiple classes of stereotypically located, ‘identified’ giant neurons in the brain, which project their large axons into the spinal cord. These giant neurons and their axonal fibers have facilitated electrophysiological recordings and imaging across biological scales, ranging from molecular to circuit-level analyses of nervous system structures and functions and including their roles in behavioral output. Second, lampreys have long been considered amongst the most basal extant vertebrates on the planet, so they have facilitated comparative studies pointing to conserved and derived characteristics of vertebrate nervous systems. These features attracted neurologists and zoologists to studies of lampreys between the 1830s and 1930s. But, the same two attributes also facilitated the rise of the lamprey in neural regeneration research after 1959, when biologists first wrote about the spontaneous, robust regeneration of some identified CNS axons in larvae after spinal cord injuries, coupled with recovery of normal swimming. Not only did large neurons promote fresh insights in the field, enabling studies incorporating multiple scales with existing and new technologies. But investigators also were able to attach a broad scope of relevance to their studies, interpreting them as suggesting conserved features of successful, and sometimes even unsuccessful, CNS regeneration. Lamprey research demonstrated that functional recovery takes place without the reformation of the original neuronal connections, for instance, by way of imperfect axonal regrowth and compensatory plasticity. Moreover, research performed in the lamprey model revealed that factors intrinsic to neurons are integral in promoting or hindering regeneration. As this work has helped illuminate why basal vertebrates accomplish CNS regeneration so well, whereas mammals do it so poorly, this history presents a case study in how biological and medical value have been, and could continue to be, gleaned from a non-traditional model organism for which molecular tools have been developed only relatively recently.

## 1 Introduction

**FIGURE 1:**
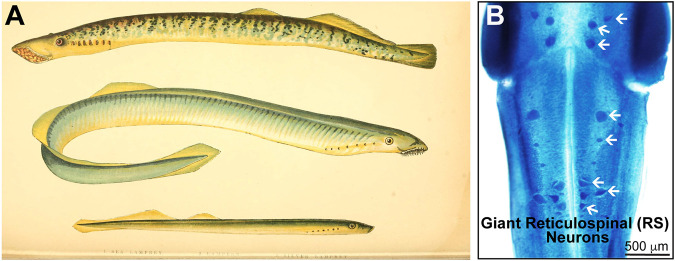
Lampreys and the giant reticulospinal (RS) neurons. **(A)** A print by natural historian Jonathan Couch (1789–1870), showing three lamprey species: (top) sea lamprey (*Petromyzon marinus*); (middle) lampern (European river lamprey, *Lampetra fluviatilis*); (bottom) silver lamprey (*Ichthyomyzon unicuspis*). These three species have predominated in neuroscience and regeneration research since the 19th century. Adapted from Couch, 1869, CCXLVII. **(B)** The brain of a larval sea lamprey stained with toluidine blue, highlighting some of the giant RS neurons in the midbrain and hindbrain (arrows). Photo credit: J. Morgan laboratory.

Since the 19th century, the lamprey (Figure 1A), a jawless fish, has been used as a laboratory organism for studies of the anatomy, physiology, and evolution of vertebrate nervous systems. Since 1959, this animal also has been employed for research into the underpinnings of central nervous system (CNS) regeneration, particularly in the spinal cord. Although not as commonly studied as more genetically tractable “model” organisms, such as zebrafish or mouse (Ankeny and Leonelli, 2021), the lamprey offers unique advantages that have perpetuated its investigation in neurobiology. In this paper, we present a history of lampreys in neurobiology from the 1830s to the present, emphasizing their evolving roles in helping to elucidate spinal cord regeneration mechanisms. Employing analyses of the scientific literature, archival documents, and interviews with scientists, our goal is not a systematic scientific review, but rather to document and explain changes and continuities over time. We end by considering some implications for biology and regenerative medicine today.

**FIGURE 2:**
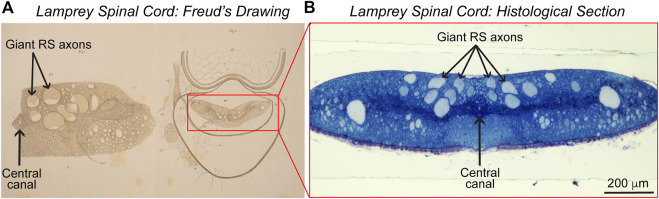
Lamprey spinal cord. **(A)** Sigmund Freud’s 1877 drawings of the lamprey spinal cord in cross-section. (Left) A drawing of half of the spinal cord, showing several giant reticulospinal (RS) axons in the ventral spinal cord and the central canal. (Right) A drawing showing the position of the spinal cord within the spinal canal. Adapted from Freud, 1877. ^©^ Freud Museum London and supplied courtesy of Freud Museum London. **(B)** Image of a lamprey spinal cord in cross-section, stained with toluidine blue. Note the similarities between the image and Freud’s early drawings. Photo credit: Emily B. Brady, J. Morgan laboratory. Ventral side is up in panels **(A,B)**.

Two attributes have long perpetuated studies of lampreys in neurobiology. First, they possess several types of large neurons in the CNS, including multiple classes of stereotypically located, ‘identified’ neurons in the midbrain and hindbrain (Figure 1B), which project their giant axons into the spinal cord (Figure 2). These giant reticulospinal (RS) neurons especially, but also the animal’s large neurons generally, have facilitated fresh insights via studies across biological scales, ranging from molecular to circuit-level analyses of neuronal structure and function employing existing and new technologies. Second, lampreys have long been understood as some of the most basal extant vertebrates on the planet—their lineage diverged from jawed vertebrates around 500 million years ago (Smith et al., 2013)—and consequently they have facilitated comparative studies pointing to conserved and derived characteristics of vertebrate nervous systems (Pombal et al., 2009; Sugahara et al., 2017; Suryanarayana et al., 2022).

In the first two sections below (the 1830s–1880s and 1860s–1930s), we show how these features initially attracted neurologists and zoologists to studies of lampreys. For example, we show how large fibers in the lamprey CNS enabled investigation of whether neurons even existed, and how lampreys figured into early debates about classification and vertebrate evolution. Then, in the following three sections, we document how the same attributes facilitated the rise of lampreys in spinal cord regeneration research after 1959, when biologists first wrote about the spontaneous, robust regeneration of some of the identified RS axons in larvae after injuries, coupled with recovery of swimming behaviors. Examined with prevailing and new laboratory technologies, not only did these large neurons enable fresh insights into how axon regrowth (1960s), compensatory plasticity (1970s–1980s), and intrinsic molecular factors (1990s–present) contribute to functional recovery, but investigators also could attach a broad scope of relevance to their studies, interpreting them as suggesting conserved features of successful (and sometimes even unsuccessful) CNS regeneration.

**FIGURE 3:**
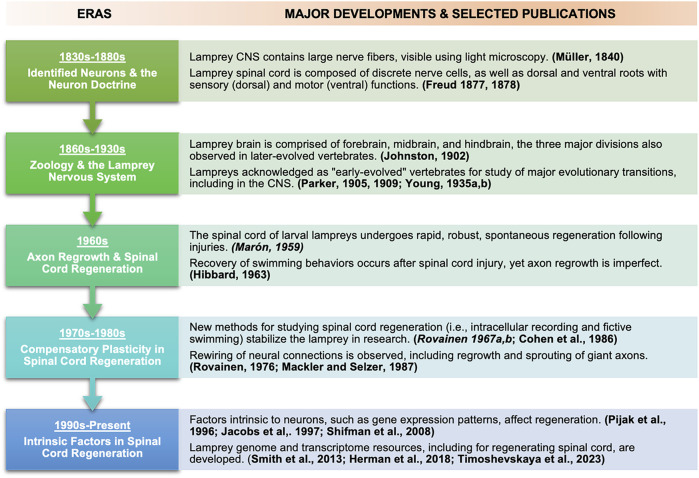
Timeline showing major eras and selected associated publications in lamprey neuroscience and regeneration research focused on spinal cord regeneration. Selected publications associated with each era are bolded. Since history does not lend itself to neat categorization, at some points major publications are listed and discussed in one time period but appeared in another (earlier) period, influencing later developments. Examples of such publications appear in *italics*.

Indeed, mammals such as humans possess only limited capacities for CNS regeneration. In large part because research in “lampreyology” helped illuminate how and why basal vertebrates accomplish CNS regeneration so well, whereas mammals do it so poorly, from 1959 onward, studies of lampreys quickly took root in medical as well as biological institutions. The lamprey’s story in CNS regeneration research is thus a history of biology and medicine. It shows how both biological and medical value have been, and could continue to be, gleaned from a non-traditional “model” organism, one for which molecular genetics tools have emerged only relatively recently. Figure 3 summarizes major developments and selected publications in the use of lampreys for neurobiology in the five historical eras we describe, focused on spinal cord regeneration.

## 2 Large nerve fibers and the Neuron Doctrine, 1830s–1880s

There are nearly 40 species of lampreys living in temperate regions across the globe.^1^ Of these, the species used most often in neuroscience research are the sea lamprey (*Petromyzon marinus*), European river lamprey (*Lampetra fluviatilis*), and silver lamprey (*Ichthyomyzon unicuspis*) (Figure 1A), with their relative use likely reflecting a combination of their availability for collection and the research locations of neuroscientists. Lampreys have an atypically long lifecycle compared to other fishes. After early embryonic development, which follows a similar progression as in other fishes, lampreys remain as slow-growing larvae (called “ammocoetes”) for 5–7 years before transforming into adults (Piavis, 1961; Tahara, 1988). Only half of known lamprey species are parasitic. As juvenile adults, these fish become parasitic and blood-feeding, and from that point on they rely on circular sets of sharp teeth for survival (Silva et al., 2013). After another 1–2 years, lampreys spawn and die, completing the life cycle.

The lamprey has long been relevant to human affairs, both as a food source and an ecological nuisance. Since at least the Middle Ages, humans have relied on lampreys as sources of food (Hoffman, 1996). King Henry I, who famously loved dining on these creatures, ignored the advice of his doctor not to indulge in the period leading up to his death, perhaps contributing to his demise (Hollister, 2001). Queen Elizabeth II was served lamprey pies, a British delicacy, at her Golden Jubilee and other anniversaries, a tradition that is expected to continue with King Charles III.^2^ Lampreys also are renowned, or perhaps reviled, as parasites. Sea lampreys feed aggressively on trout, salmon, and other large game fish, causing serious impacts on fishing in the U.S. Great Lakes: Only one of seven fish attacked by a lamprey will survive.^3^ For much of the last century, considerable public funding thus was allocated to the eradication of the sea lamprey, for instance towards research into the animal’s lifecycle.

Additionally, lampreys are studied in many fields of life science. The sea lamprey, *Petromyzon marinus*, was named by the systematist Carl Linnaeus in 1758. 19th-century zoologists pondered where lampreys belonged in relationships between organisms reflecting debates over species classification and evolutionary theories (Bowler, 1996; Blackman, 2007). Today, many species of lampreys are variously used in biological studies on topics as wide-ranging as evolutionary-developmental (evo-devo) biology (Green and Bronner, 2014; York and McCauley, 2020), adaptive immunity (Boehm et al., 2018; Das et al., 2021), endocrinology (Sower and Hausken, 2017), and ecology (Cuhel and Aguilar, 2013). Moreover, it was in the hands of a young Sigmund Freud, a newly minted doctor, that lampreys made one of their earliest splashes in cellular neurobiology. In the 1870s and 1880s, Freud carried out a series of studies in neurology, or the study of (especially vertebrate) neuroanatomy for medical purposes, that proved foundational to his famed development of psychoanalysis in the 20th century (Guenther, 2012, 2015). Yet, the property that drew Freud to lampreys was not their capacity for CNS regeneration. Rather, Freud was attracted to analyses of their large neurons, which proved accessible with the reigning methods of dissection, fixation, staining, and light microscopy (Shepherd, 2016).

It had been known since at least the 1830s work of the Berlin anatomist, Johannes Müller, that the lamprey CNS contains prominent neurons (Müller, 1840). One example was the dorsal cells in the spinal cord, today understood as perhaps homologous with Rohon-Beard cells in jawed fishes (Johnels, 1958; Rovainen, 1967b). Also known in the 19th century were what we now call “identifiable” neurons—where neuronal cell bodies (somata) and axonal fibers remain in stereotypical locations across generations—although identifiable axons today are thought to be more common in invertebrates, the classic example being the squid giant axon (Young, 1936). In the lamprey, for example, we now understand the ‘Müller neurons’ to be the giant reticulospinal (RS) neurons in the midbrain and hindbrain, which measure up to several 100s of μm in diameter (Figure 1B). The “Müller fibers” are the giant RS axons which emanate from “Müller neurons”; they are 20–60 μm in diameter and traverse the ventromedial tract of the spinal cord (Figure 2) (Rovainen, 1967a). Müller well may have been the first to write about these specific fibers, yet later several additional classes of large, identified neurons would be discovered in the lamprey CNS (Rovainen, 1967a; 1967b). Moreover, in Müller’s time, it was still an open and debated question whether nervous systems generally consist of bounded nerve cells, with membranes and somata connected to axons, or of continuous, interconnected syncytia. The former argument came to be known as the Neuron Doctrine, supported famously by the Spaniard Santiago Ramón y Cajal (Jones, 1994, 1999; Shepherd, 2016). Relatedly, and especially after the publication of Charles Darwin’s *On the Origin of Species* in 1859 (Darwin, 2009), anatomists wondered when in time nerve cells had evolved, if they existed, and how neural architecture differs and compares in vertebrates and invertebrates (Anctil, 2015).

Like most dons of 19th-century German anatomy, Müller taught a large cadre of students (Otis, 2007), and it was in this context that his lamprey research was passed down to succeeding generations. Having graduated with his medical doctorate from the University of Vienna in 1877, Freud soon found himself seeking research experience under Ernst von Brücke, Müller’s former student (Shepherd, 2016). Freud had grown interested in neurophysiology through visits to the zoological station in Trieste, founded in 1875 in part to supply experimental organisms to the universities in Vienna and Graz (Zavodnik, 1995; Gandolfi, 2010). Freud went to work with Brücke to study an enigmatic fibrous structure in the lamprey spinal canal, discovered in 1860 by the anatomist Ernst Reissner (Reissner, 1860). By 1877, neither the developmental origins nor the functions of “Reissner’s fiber” had been elucidated, and Brücke wanted Freud to clarify these points. It was by way of this work that Freud also came to draw several conclusions supporting the Neuron Doctrine, an outcome that perpetuated research on the lamprey CNS.

The historian-neuroscientist Gordon M. G. Shepherd has argued that, while tracing the large fibers of the dorsal cells found in the peripheral nervous system (PNS) to their cell bodies in the dorsal root of the lamprey spinal cord, Freud was under the impression that one of these somata gave rise to Reissner’s fiber (Shepherd, 2016). Thus, Freud hypothesized that the latter was in fact an axon. Today, there is no broad agreement on the function of Reissner’s fiber (Cantaut-Belarif et al., 2018; Driever, 2018; Aboitiz and Montiel, 2021): It is generally understood to be a conserved, proteinaceous structure synthesized during development by the subcommissural organ, located in the dorsocaudal diencephalon just below the posterior commissure. It is therefore not an axon, as Freud thought; one hypothesis is that the fiber is involved “in the origin of fundamental innovations of the chordate body plan, especially the elongation of the neural tube and maintenance of the body axis” (Aboitiz and Montiel, 2021). Yet, history is full of such productive 'mistakes': points where interpretations later deemed incorrect have nonetheless led to other work that has stood the test of time (Kaiser and Creager, 2012).

In his two papers examining the sea lamprey (*Petromyzon marinus*) and the European brook lamprey (*Petromyzon planeri,* now renamed *Lampetra planeri*), Freud traced the large sensory fibers now understood to belong to the dorsal cells from their origins in the PNS into the dorsal root of the spinal cord (Freud, 1877, 1878).^4^ His 1877 drawing of half of the spinal cord, in turn, shows several giant reticulospinal (RS) axons in the ventral spinal cord and the central canal (Figure 2A). Based on this histological work, Freud concluded that the dorsal (“posterior”) and ventral (“anterior”) roots of the cord are responsible for sensory inputs and motor outputs, respectively. He also emphasized that these roots are composed of discrete nerve cells, consisting of soma and fiber conjoined, and believed that the lamprey’s dorsal cells represented an evolutionarily transitional form from invertebrates to higher vertebrates (Shepherd, 2016). In 1882, Freud continued this line of work in the freshwater crayfish, enriching his conclusions about neurons (Freud, 1882). That vertebrate spinal cords are composed, structurally, of dorsal and ventral roots with different functions remains a tenet of neuroanatomy. Of course, the notion that nervous systems are largely cellular and comprised of discrete neurons also has persisted, even as the Neuron Doctrine itself has undergone historical revisions (Guillery, 2005, 2007; Shepherd, 2016; Maxson, 2021).

## 3 Zoology and the lamprey nervous system, 1860s–1930s

Neurology hardly represented the only avenue by which 19th-century biologists investigated lampreys, however. Zoologists also turned to studies of these animals, attempting to situate them within contemporary species classifications and emerging evolutionary theories of life. By the 1930s, when Darwin’s notion of evolution by natural selection had grown broadly accepted, lampreys emerged as organisms well-suited for comparative studies, including those attempting to elucidate conserved and derived features of vertebrate nervous systems.

Across the 19th century, for example, zoologists pondered where lampreys belonged in relationships between organisms reflecting heated debates over classification systems and evolutionary theories (Bowler, 1996; Secord, 2000; Blackman, 2007). Jonathan Couch, a respected doctor-turned-zoologist, published several books systematically documenting the fishes of the British islands, including lampreys (Figure 1A) (Couch, 1868, 1869). Couch likely subscribed to some combination of Linnaean and other classification systems prominent at the time (Naylor, 2005), and in 1868 he noted of lampreys that they occupied “the vanishing point of fishes in their transition towards the class of worms” (Couch, 1868). By later in the century, broad acceptance of Darwin’s theory of evolution was coupled to general agreement that the characteristics of lampreys were likely to be conserved rather than degenerate (Bowler, 1996; Blackman, 2007). In turn, prominent zoologists, such as Thomas Henry Huxley, encouraged studies of lampreys alongside other creatures, such as sharks, that seemed to offer insights into the evolution of vital processes (Blackman, 2007).

Collecting and studying wild organisms, such as sharks and lampreys, also allowed zoologists to escape the doldrums of dry indoor laboratories (Ericson, 2020; Luk, 2020). Zoologists frequently accompanied Royal Navy ships to collect specimens for study (Rozwadowski, 2008). A related development promoting comparative zoology was the “station movement,” or the 19th-century appearance of hundreds of coastal laboratories for the study of oceanography, fisheries development, and biology (Muka, 2014; de Bont, 2015; Matlin et al., 2020; Maxson, 2021). Across the century, many European zoologists flocked to shorelines for the diverse flora and fauna they could encounter there (Jack, 1945). One such biologist was Anton Dohrn, an ardent follower of Darwin who went on to establish the *Stazione Zoologica* in Naples, Italy in 1872 (Dohrn, 1872; Groeben, 2020). Other such stations followed, such as that at Trieste, the Laboratory of the Marine Biological Association of the United Kingdom in Plymouth, England (f. 1884), and the Marine Biological Laboratory (MBL) in Woods Hole, Massachusetts (f. 1888) (Maienschein, 1985; Monroy and Groeben, 1985; Erlingsson, 2009), promoting the study of marine creatures and often aspects of their neurophysiology.

It was in this context that John Zachary Young, the author of the quote from which the subtitle of this article is taken, turned to studies of lampreys. Young completed his education at Oxford in 1928, where he read zoology, neurophysiology, and some comparative anatomy (Young, 1996). In 1929, Young began a fellowship at the *Stazione* under the Italian physiologist Enrico Sereni, commencing investigations of the autonomic nervous systems of fishes and degeneration and regeneration in octopus’ pallial nerves (Sereni and Young, 1932; De Leo, 2008; Imperadore and Fiorito, 2018; De Sio and Imperadore, 2023). Young soon also grew interested in the lateral line nerves of fishes, understood today to be sensory systems involved in vibration and motion detection. Additionally, he was curious about the pineal and pituitary glands, about which little was known for most vertebrates. Suspecting involvement of the pineal gland in photoreception, Young began examining lampreys from the lakes around Oxford and Worcester in the 1930s, publishing two papers in 1935 that helped solidify lampreys as well-suited for comparative studies probing conserved features of nervous systems (Young, 1935a; 1935b). Before Young, George Howard Parker, a Harvard zoologist, had studied lamprey photoreception, utilizing animals sent from New York to Massachusetts (Parker, 1905, Parker, 1909). Parker had suggested that the tails of larval lampreys (ammocoetes), and perhaps of all fishes, were light sensitive, “since so primitive a fish as ammocoetes exhibited this peculiarity” (Parker, 1909). Parker soon concluded that such sensitivity did not extend to all fishes, although he did suggest that further studies would be informative. Two and a half decades later, Young found that the pineal gland in ammocoetes played a “leading part in controlling the colour of the animal” in response to light, maybe even “in regulating other and still more significant functions of the pituitary” (Young, 1935b). “Even at this early stage of its (evolutionary) history,” Young suggested, “the pineal complex is connected not so much with somatic as with visceral functions” (Young, 1935b).

Young’s findings also agreed with the dense work that had been emanating from neurologists in Europe and the United States since at least the 1890s (Shepherd, 2016), establishing that the lamprey CNS was indeed a highly illustrative transitional form. For instance, as the University of West Virginia neurologist, John Black Johnston, published in his “attempt to define the primitive functional divisions of the central nervous system” in *Petromyzon* in 1902, the lamprey brain comprised all three divisions also observed in later-evolved vertebrates, the forebrain, midbrain, and hindbrain, alongside what was called the “tween brain” the diencephalon now considered part of the forebrain (Johnston, 1902; other references reviewed in, Pombal et al., 2009).

These early studies also led to the conclusion that lampreys and hagfish lacked several higher order brain structures seemingly acquired after the cyclostomes (jawless vertebrates) split with the gnathostomes (jawed vertebrates). However, more recent molecular and physiological work has revealed that lampreys and hagfish do possess these structures (e.g., medial ganglionic eminence, rhombic lip) (Sugahara et al., 2017; Sugahara et al., 2022), and that the lamprey forebrain in fact displays functional connections and neuronal subtypes observed in the larger mammalian neocortex (Grillner and Roberson, 2016; Grillner, 2021a; Suryanarayana et al., 2022). An updated prosomeric model of the lamprey brain suggests further commonalities of forebrain development and architecture with the gnathostomes (Pombal and Puelles, 1999; Pombal et al., 2009). Thus, the current view is that lampreys and hagfish possess a “blueprint” of the vertebrate brain, already present in the ancestral vertebrate (Sugahara et al., 2017; Grillner, 2021a). The leading model of vertebrate evolution still supports cyclostome monophyly, or the argument that lampreys and hagfish form a clade on the same branch (Miyashita et al., 2019; Kuratani, 2021), and any revision to the current model awaits additional molecular insights or updates from the fossil record.

Even by the 1930s, however, the lamprey was collecting an increasing number of roles as an experimental organism in neurobiology. It displayed large neurons in the brain, which were possible to view with existing microscopy techniques. It also had features that, even at the time, were believed to illuminate transitions in CNS evolution: “so many of our own (mammalian) mechanisms,” J.Z. Young later exclaimed, “in a less elaborate condition” (Hardisty and Potter, 1971.) For these reasons, the lamprey held “a very special claim on the interest of zoologists,” with Young initially hoping that elucidating its complete cellular wiring and developmental stages might clarify “the whole life systems of the animals in relation to their environment” (Hardisty and Potter, 1971). Of course, this dream never transpired, at least not in Young’s lifetime. This was in part because the cellular anatomy was simply too hard to work out before the spread of electron microscopy in the 1950s (Rasmussen, 1997). Also, it was only by way of modern molecular techniques that some definitions and comparisons of brain structures, regional borders, and neuronal subtypes between lampreys and other vertebrates became possible. After Young’s ascendancy to the Chair of Anatomy at University College, London (UCL) in 1945, his own work turned largely to higher vertebrates and cephalopods (De Sio, 2011, 2018).

## 4 Axon regrowth and spinal cord regeneration in lampreys, 1960s

After World War II (WWII), studies of lamprey neurobiology hit yet another turning point: in 1959, lamprey research entered and began proliferating in studies of spinal cord regeneration. Examined with prevailing and new laboratory technologies, as we show in this and the following sections, not only did giant lamprey RS neurons enable fresh insights into how axon regrowth (1960s), compensatory plasticity (1970s–1980s), and intrinsic molecular factors (1990s–present) contribute to the recovery of function, understood as the recovery of normal swimming. But investigators could also attach a broad scope of relevance to their studies, interpreting them as suggesting conserved features of successful regeneration. Because this work showed promise in illuminating how and why basal vertebrates accomplish CNS regeneration so well, whereas mammals fare so poorly, it also began taking root in medical as well as biological research.

As K. Marón, a biologist at the Department of Experimental Zoology at the Polish Academy of Sciences in Kraków, noted in 1959: “Up to now,” the neurobiological community had not seen “any works in literature treating of the regeneration capacity of the central nervous system in cyclostomes (lampreys and hagfishes)” (Marón, 1959). “The evolutionary significance” of these organisms made this gap problematic, so Marón set out to record tissue healing following spinal cord injury in the ammocoetes of *Lampetra fluviatilis* (European river lamprey) using light microscopy (Marón, 1959). He documented the formation of a bridge of ependymal cells (of glial origin) in the transection site after around 5 days (Marón, 1959). He also reported that “after 20 days both severed ends of the cord are … joined by numerous nerve fibres,” such as what appeared to be the giant Müller fibers (Marón, 1959). This study provided a general descriptive framework for neural regeneration in the CNS of a basal vertebrate.

Soon thereafter, Emerson Hibbard, a neurobiologist at the US National Institute of Neurological Disorders and Stroke (NINDS), built on Marón’s work. Hibbard employed no cutting-edge technologies. Rather, light microscopy and common tissue fixation methods gained new power when applied to the large, identified neurons of *Petromyzon marinus*, enabling unprecedented precision in correlating the regrowth of axons with tissue repair and behavioral recovery. “Ordinarily the spinal cord appeared to be essentially normal by 20 days after being severed,” Hibbard observed (Hibbard, 1963). The “giant axons had traversed” the injury site (Hibbard, 1963). The “ability of the animal to perform coordinated sinuous movements of the trunk and tail posterior to the wound when *out* of water was taken as the criterion for functional recovery” from the injury, with normal swimming returning after around 20 days (Hibbard, 1963). Hibbard understood this spontaneous recovery to be at least partly caused by the regrowth of the giant fibers, and he pointed to the relative speed with which functional recovery occurred in lampreys (Hibbard, 1963). Moreover, Hibbard’s work clearly was motivated by a combination of the convenience of working with large neurons and the hope that, through comparison and contrast, features of lamprey spinal cord regeneration could shed new light on why mammals fared so poorly in this regard. “The difficulty in obtaining good functional neural regeneration within the spinal cords of higher vertebrates, and especially of man, has caused many investigators to focus their attention … in lower forms, particularly in fish and amphibians,” he wrote in the opening lines of his paper in 1963 (Hibbard, 1963).

By the early 1960s, nearly two centuries of research had made it clear that spontaneous and robust neural regeneration occurs readily in the CNS across the animal kingdom, except in mammals. 18th-century naturalists had studied tail regeneration in lizards, a process involving the CNS (Spallanzani, 1768; Dinsmore, 1991; Tsonis and Fox, 2009). 19th-century biologists had examined the robust structural and functional regeneration that takes place in the CNS of invertebrates and the mammalian PNS (Stahnisch, 2003, 2016, 2022). Investigators so far in the 20th century had focused on optic nerve regeneration in frogs and toads, amphibian tail and spinal cord regeneration, chemical factors inducing nerve growth in chick embryos, spinal cord regeneration in goldfish, and nervous system regeneration in crustaceans and cephalopods (Sperry, 1943, 1945; Cohen and Levi-Montalcini, 1956; Bernstein, 1964; Clemente, 1964; Hoy et al., 1967; Larner et al., 1995; Meyer, 1998; Allen, 2004; Imperadore and Fiorito, 2018; De Sio and Imperadore, 2023). Santiago Ramón y Cajal also had examined what he called the “plastic” capacities of the mammalian CNS from the 1890s through the 1930s, concluding that while some sprouting of damaged axons was possible, this regrowth had uncertain functional relevance (Ramón y Cajal, 1928; Stahnisch and Nitsch, 2002; Stahnisch, 2003). We also now know that many non-mammalian vertebrates, such as teleosts and amphibians, undergo robust axon regeneration and functional recovery, often on even faster time frames than lampreys (Tanaka and Ferretti, 2009; Morgan and Shifman, 2014; Morgan, 2017; Hanslik et al., 2019; Cigliola et al., 2020; Alper and Dorsky, 2022).

**FIGURE 4:**
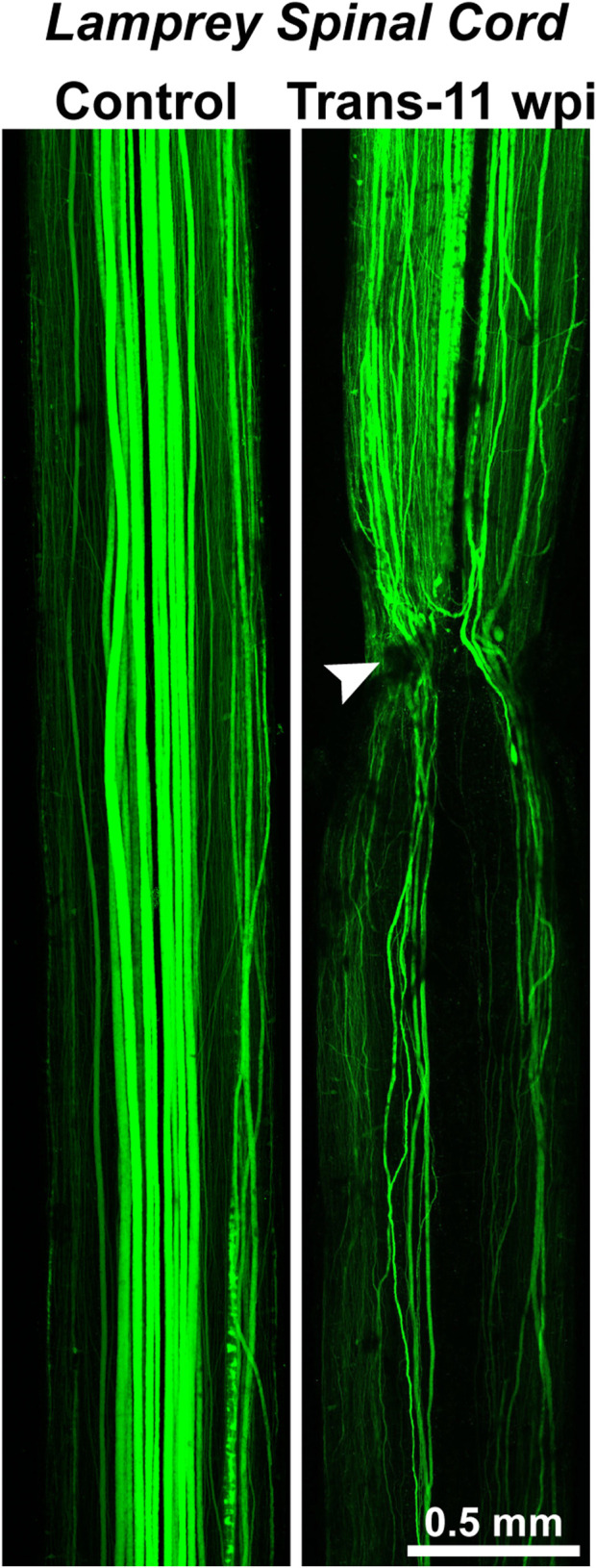
Axon regeneration in the lamprey spinal cord. (Left) Confocal projection of an uninjured, control lamprey spinal cord where the RS axons were anterogradely labeled with Alexa488-dextran. The descending RS axons typically exhibit straight, non-branching projection patterns. (Right) Confocal projection of a transected (Trans) spinal cord at 11 weeks post-injury (wpi). Anterograde labeling shows that the regenerating axons exhibit vastly different projection patterns. While some regenerating axons traverse the transection site (arrowhead) and cross into the distal spinal cord (below the transection site), other axons fail to regenerate, branch, or turn rostrally, demonstrating the imperfectness of structural regeneration. Despite this, the functional recovery of swimming behaviors is remarkably robust in these animals. Rostral is to the top. Adapted from Haspel et al., 2021 and used with permission by Wiley.

Nevertheless, “the difficulty in obtaining good functional neural regeneration within the spinal cords of higher vertebrates, and especially of man” meant biologists were seeking new organisms and methods with which to study this process and fresh insights into its underlying mechanisms (Hibbard, 1963). Of the conserved properties of CNS regeneration, Hibbard offered at least two further insights that would guide later research. First, he showed that CNS regeneration in the lamprey was far from structurally perfect. Some RS axons grew back across the injury site, but “aberrant fibers” were also “found wandering off in various directions from the wound” (Hibbard, 1963). This is clearly demonstrated in Figure 4, a modern image that shows where regenerating axons in the transected lamprey spinal cord can be observed projecting in atypical pathways relative to the uninjured control spinal cord. *Functional regeneration* thus appeared possible in the lamprey CNS, even if by way of imperfect *structural regeneration*. Second, Hibbard pointed to the significance of the *extracellular milieu*, the environment outside neurons, in either promoting or hindering CNS regeneration. “The vascular supply to the spinal cord of the lamprey indicates a complete absence of capillaries within the cord but a rich plexus of capillaries overlaying it in the meninges,” Hibbard wrote (Hibbard, 1963). The flattened shape of the spinal cord (Figure 2B) also “permits all cellular elements … to obtain necessary oxygen and metabolites by diffusion or active transport” (Hibbard, 1963). Thus, “the (lamprey) system precludes extensive destruction and atrophy of both neurons and supporting elements,” minimizing scarring (Hibbard, 1963). Hibbard, like neurobiologists today, interpreted these features as pro-regenerative, in contrast to in the mammalian CNS, where “disturbance of the vascular bed, the resultant atrophy of cells, and the necessity for removal of breakdown products” leads to scarring that hinders self-repair (Hibbard, 1963).

Both imperfect structural regeneration and the role of the extracellular milieu in spinal cord regeneration were attracting attention in other contexts. For instance, Jerald Bernstein at the National Institute of Neurological Diseases and Blindness (NINDB) wrote in 1964 that in larval goldfish, “normal swimming returned” following spinal cord injury, despite the failure of many fibers to regenerate (Bernstein, 1964). Soon, similar findings were observed in zebrafish, axolotls, and newts (Bernstein and Gelderd, 1970, 1973). Additionally, Carmine D. Clemente, William F. Windle, and William W. Chambers at the University of Pennsylvania School of Medicine had been arguing that a drug, Pyromen, could block the activity of astrocytes and the formation of glial scars in adult cats and dogs, apparently allowing for modest axonal sprouting in the CNS (Windle and Chambers, 1950; Windle et al., 1952; Clemente and Windle, 1954; Clemente, 1964). By the mid-1960s, therefore, new optimism about probing and promoting functional CNS regeneration in humans was blossoming, spurred by yet further technological developments rapidly infiltrating neurobiology, such as electron microscopy (Palade and Palay, 1954; Palay, 1956; Gray, 1959) and novel techniques for staining the injured mammalian cortex (Nauta and Gygax, 1954; Stahnisch, 2003). This also was true despite centuries of research having garnered pessimism, and many mechanistic details remaining unclear, including of the properties of successful CNS regeneration at the levels of the neuron and below and of what hinders and promotes this process in mammals (Stahnisch, 2022).

“It is now entirely reasonable to abandon the view that central nervous regeneration cannot be accomplished in man,” Lloyd Guth and William Windle, neurologists at NINDS and New York University, declared at a conference in 1970 (Guth and Windle, 1970). Furthermore, as the “regeneration of axons, including Mauthner fibers, in the severed spinal cord of the chordate larval lamprey” occurred so readily, “one key to unlock the secrets of the enigma [of CNS regeneration] may lie here” (Guth and Windle, 1970).

## 5 Compensatory plasticity in spinal cord regeneration in lampreys, 1970s–1980s

In the 1970s and 1980s, lamprey research continued to gain traction within the field of CNS regeneration, still focusing on the neurons of the spinal cord after traumatic injury. This time employing new technologies, researchers again expanded their scales of analysis, accumulating insights especially into an attribute related to imperfect structural regeneration: “compensatory plasticity,” or the rewiring of neural networks to achieve functional recovery.

**FIGURE 5:**
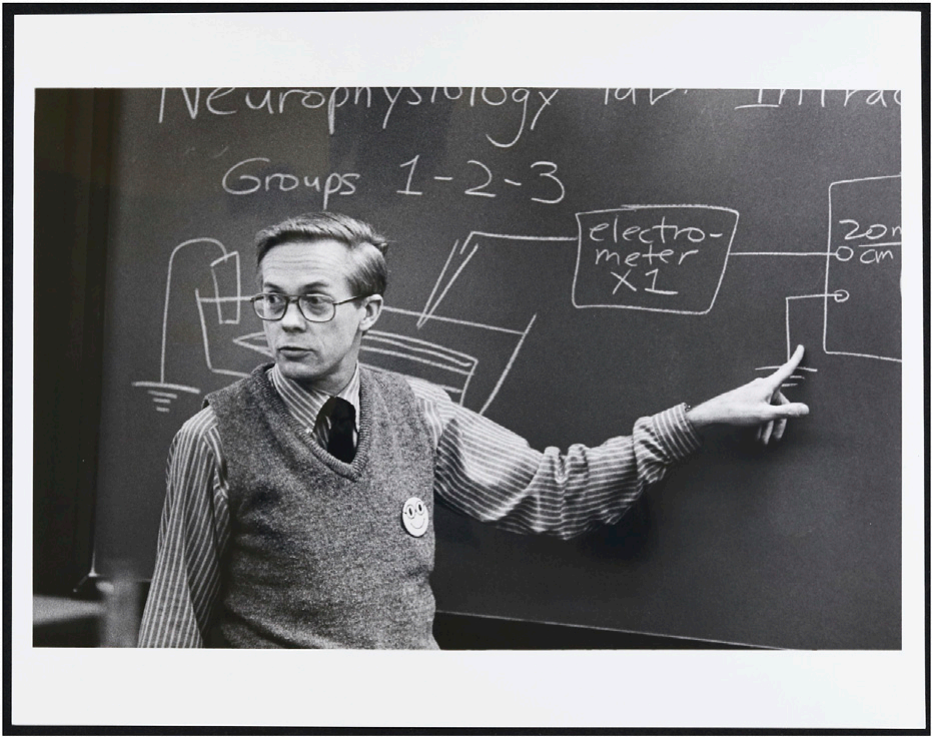
Dr. Carl Rovainen. Dr. Carl Rovainen giving a lecture, Department of Cell Biology and Physiology, Washington University School of Medicine, circa 1983. Used with permission by Becker Medical Library, Washington University School of Medicine.

In 1964, a young neurophysiologist, Carl Rovainen (Figure 5), began studying the lamprey while working on his Ph.D. at Harvard Medical School. During a stint one summer at the nearby MBL in Woods Hole, Steven Kuffler, who would go on to establish the Harvard Department of Neurobiology in 1966, suggested that Rovainen work on the lamprey (McMahan, 1990). Kuffler and John Nicholls, also at Harvard, were studying the leech, and they had “sought a vertebrate counterpart to the leech preparation of neurons and glia” (McMahan, 1990). The pair had “made the first unpublished intracellular recordings from large Müller neurons in the lamprey brain,” Rovainen remembered later, “but because they could not record also from glial cells, they asked me, as a graduate student in need of a project, whether I would like to continue the recordings from the large nerve cells” (McMahan, 1990).

Rovainen agreed, becoming the first to characterize the functions of the identified giant RS neurons in the lamprey brain. Alan Hodgkin and Andrew Huxley had been the first to record neuronal voltages internally, employing squid giant axons (Hodgkin and Huxley, 1939, 1945). Similar invertebrate studies had followed (i.e., Hodgkin and Keynes, 1953), including employing pre- and postsynaptic electrodes in the squid giant synapse (Bullock and Hagiwara, 1955, 1957). By the 1960s, intraneuronal recordings also had been carried out in dog and goat Purkinje fibers, cat motor neurons, and Mauthner neurons in various fishes (Draper and Weidmann, 1951; Brock et al., 1952; Tasaki et al., 1954; Furshpan and Furukawa, 1962; Furukawa et al., 1963; Furukawa and Furshpan, 1963; Auerbach and Bennett, 1969). Yet, the sizes of the lamprey’s RS neurons, some of them several times larger than even catfish and hatchetfish Mauthner neurons, made the lamprey an attractive animal for which to develop intracellular methods. In 1967, using serial sectioning and a variety of intracellular and extracellular stimulations and recordings, Rovainen identified the functions and synaptic connections of many of the large, identified neurons in sea lamprey, as well as of the dorsal cells and large interneurons (Rovainen, 1967a; 1967b). He even documented the associations of some cells with specific movements, such as tail flexions, body rotations or contractions, and fin movements. In 1967, Rovainen took up a faculty position in the Department of Physiology and Biophysics at Washington University in St. Louis, where he remained until retirement in 2001.^5^ While his watershed papers on the functions of the identified neurons had not addressed regeneration, in the 1970s Rovainen also took up studies of this phenomenon.

**FIGURE 6:**
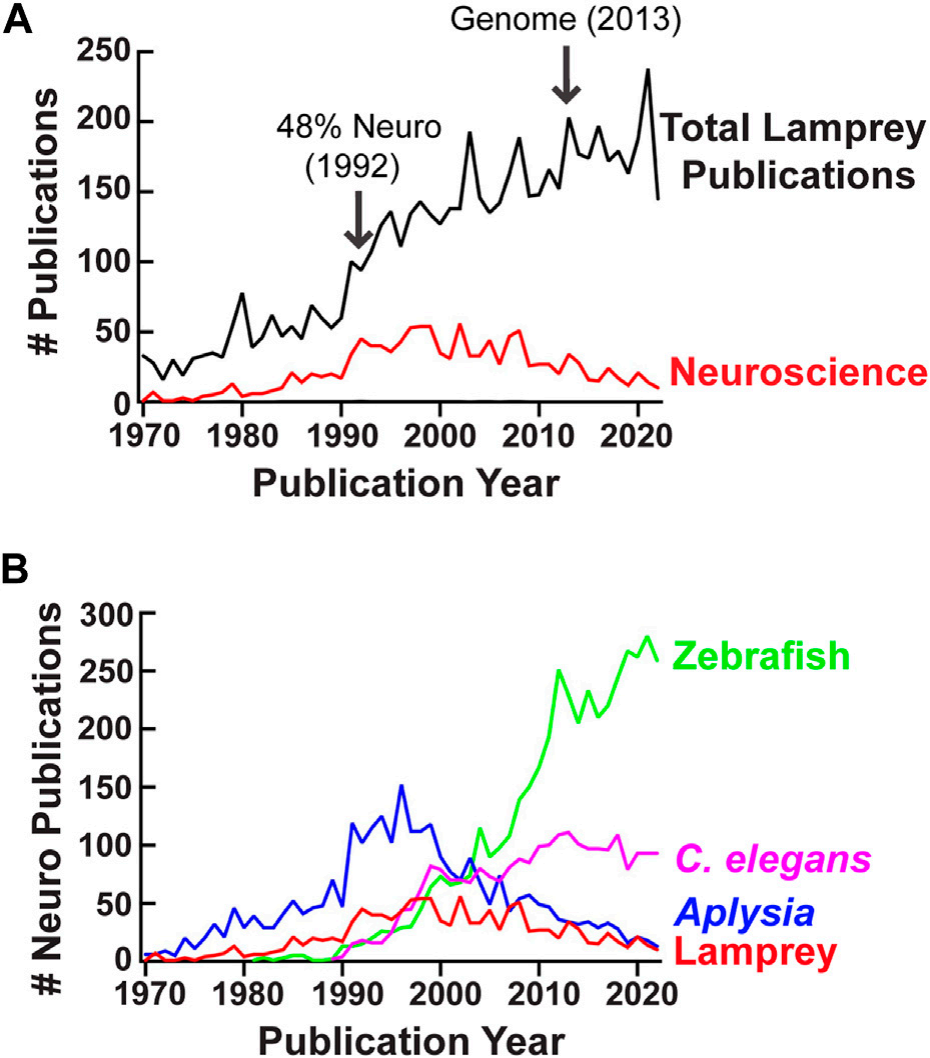
The lamprey as an experimental organism in neuroscience. **(A)** The total number of journal articles, book chapters, and review articles in ISI Web of Science using lampreys as a model organism, and the number in neuroscience, 1970-2022. The total number has increased steadily since the 1970s. However, while neuroscience publications comprised approximately half of the total lamprey journal articles, book chapters, and review articles in the mid-1990s (peaking at around 48% in 1992), that proportion has declined in recent decades (see Supplementary Material S1). (**B)** The numbers of neuroscience journal articles, book chapters, and review articles in ISI Web of Science for lamprey, *Caenorhabditis elegans*, *Aplysia*, and zebrafish, 1967-2022. A decline of lamprey neuroscience publications in the late 1990s coincided with a sharp rise for zebrafish and *Caenorhabditis elegans*. These trends are similar across the four organisms shown for neuroscience publications normalized to all neuroscience journal articles, book chapters, and review articles, 1967-2022 (see Supplementary Material S1). Methods for generating the graphs in Panels **(A,B)** can be found in Supplementary Material S1.

It is around this turning point, in the early 1970s, that the number of total lamprey publications began to increase dramatically, bolstered in part by the entry of Carl Rovainen and others into the study of lamprey neuroscience. In addition, a 1971 book, *The Biology of Lampreys*—in which J.Z. Young reflected on how lampreys had been, and could be, employed as experimental organisms—perpetuated studies by assessing and consolidating the knowledge to that date (Hardisty and Potter, 1971). The total number of journal articles, book chapters, and review papers published each year employing lampreys has increased steadily from the 1970s to the present day (Figure 6A). Those in the “neurosciences” category designated by ISI Web of Science also increased from the 1970s until the mid-1990s, stabilizing in the early 2000s at a time when neuroscience journal articles, book chapters, and review papers employing genetic model organisms, such as zebrafish and *Caenorhabditis elegans*, were rapidly increasing (Figure 6B). This growth can be attributed in large measure to influential researchers such as Rovainen. Another such scholar is Professor Sten Grillner, the Director of the Nobel Institute for Neurophysiology since 1987 (Grillner, 2021b). The collective works from Grillner and his colleagues have helped to clarify how neural networks are organized in the lamprey, how they control locomotor and sensory behaviors, and, more broadly, how the vertebrate CNS evolved (McClellan and Grillner, 1983; McClellan, 1984; Pombal and Puelles, 1999; Grillner, 2006, 2021a; Grillner and Wallén, 2006; Grillner and Robertson, 2016). Michael Selzer, Avis Cohen, and their colleagues have had similar impacts on the use of lampreys for studying neural networks controlling locomotor functions and mechanisms of CNS regeneration, as will be discussed later (Cohen, 2019; Selzer, 2019).

Reflecting this community growth, in 1976 Rovainen published a paper that built on Marón and Hibbard’s conclusions from the previous decade and stimulated a wave of research on compensatory plasticity induced by spinal injury (Rovainen, 1976). Employing thousands of serial sections, Rovainen showed that despite recovery of normal swimming, Müller neurons in the sea lamprey were only somewhat competent at regeneration. Additionally, while some of the descending RS axons typically regenerated, others, such as the Mauthner neurons, did not. Of his findings, Rovainen remarked: “the substantial behavioral recovery after spinal regeneration despite obvious morphological abnormalities is surprising both in lampreys and in other lower vertebrates” (Rovainen, 1976). “The most important mechanism for functional recovery,” he continued, “may (thus) be morphological and physiological alterations which amplify the actions of the fewer descending axons” (Rovainen, 1976): that is, the re-arrangement of neuronal networks.

Several groups soon extended these results, suggesting that functional recovery indeed took place at least partly through the rewiring of neural circuits, rather than just the reformation of the original neuronal connections. For instance, Michael Selzer, who was then on faculty at the University of Pennsylvania School of Medicine, determined that RS neurons and other spinal neurons in the larval sea lamprey could re-establish electrophysiological activity across the lesion site, as measured by intracellular recordings (Selzer, 1978). Yet, new axonal sprouting and synapse formation also helped bring about recovery. Selzer’s group, and Malcolm Wood and Melvin Cohen in the Yale Department of Biology, confirmed these conclusions, employing intracellular injections of horseradish peroxidase into regenerating axons to visualize the new axon sprouts and newly formed synapses (Wood and Cohen, 1981; Yin and Selzer, 1983). These studies revealed a substantial amount of aberrant axonal sprouting and projection patterns, as well as incomplete axonal regrowth in the regenerating lamprey spinal cord (Figure 4), despite robust recovery of swimming behaviors.

Then, during the 1980s, researchers continued to investigate compensatory plasticity in lampreys. In 1980, during her transition from the Karolinska Institute (where she worked with Grillner) to Biological Sciences at Cornell University, Avis Cohen helped develop a method to induce the rhythmic activity of the motor neurons underlying swimming in the dissected larval sea lamprey spinal cord, using a bath application of D-glutamate or L-DOPA (Cohen and Wallén, 1980). Ventral root activity could then be studied *in vitro*. Cohen, Selzer, and Scott Mackler later employed this “fictive swimming” methodology to show that a lamprey’s regenerated CNS axons incorporated into the central pattern generator (CPG) networks for swimming, giving rise to motor neuron activity with a high degree of phase-locking across the lesion site (Cohen et al., 1986). The axonal connections, in other words, gave rise to normal electrical patterns despite aberrant sprouting and their new synaptic connections. This was a watershed contribution to the field because it demonstrated, in a vertebrate, that regenerating descending spinal axons played an important role in coordinating entire neural networks for locomotion during functional recovery from spinal injury. In 1987, Mackler and Selzer confirmed that despite the aberrant regrowth patterns, regenerating RS axons nonetheless exhibited some selectivity in choosing postsynaptic partners, finding their ways to the same subtypes of spinal motor neurons or interneurons as in the uninjured spinal cord (Mackler and Selzer, 1987).

As the 1980s progressed, the lamprey thus had been solidified in biological and medical institutions, from marine laboratories and biology departments to medical schools, as an organism for which presumably conserved features of CNS regeneration, including compensatory plasticity, could be studied at multiple scales. “Plastic” phenomena certainly held general interest: Several groups had been examining the re-wiring of invertebrate neural networks for learning, for instance, as the funding and institutional infrastructures for neuroscience grew rapidly in the decades following WWII (for the infrastrucrues: Schmitt et al., 1975; De Sio, 2018; Maxson Jones, 2020; Prkachin, 2021; for studies of invertebrate learning: Kandel and Tauc, 1964, 1965; Alkon, 1973, 1983; Kandel, 1976). Yet, in no other vertebrate besides the lamprey could electrical activity and behavioral changes be correlated with axon regeneration and the rearrangement of neural networks with such precision, an assertion that holds to the present day. Studies of compensatory plasticity also had the potential to reframe the end goals of therapies for CNS injuries and diseases: if the phenomenon took place in mammals, as it seemed to do in research with rodents (Barker and Eayrs, 1967; Miller and Lund, 1975; Barlow and Gaze, 1977; Lund, 1978; Kiernan, 1979, 157; Stahnisch, 2022), then therapies could focus on promoting new functional states, rather than on restoring all the original neuronal connections.

## 6 Intrinsic factors in spinal cord regeneration, 1990s–Present

As the 1990s dawned, lampreys certainly held a unique position in neurobiology, not only in studies of CNS regeneration but also in research on compensatory plasticity more generally. However, despite the considerable public and private funding allocated towards “regenerative medicine” since at least the 1970s (Maienschein, 2011), frustration was mounting in scientific and clinical communities regarding a lack of translation of research findings into medical therapies. On the one hand, as a lengthy review by John Kiernan in the Department of Anatomy at the University of Western Ontario made clear in 1979, much previous research on mammalian CNS regeneration had turned out to be plagued by serious limitations. For example, Pyromen, the drug that in the 1950s had appeared to promote CNS regeneration in adult cats and dogs, had proven short-lived in its promising results (Kiernan, 1979). Moreover, it appeared that many of even the most careful spinal cord transections in rats were incomplete, leaving some spinal tissues intact and thus providing 'bridges' across which sprouting uninjured axons could grow, muddying the relevance of those studies for developing future medical therapies targeting CNS regeneration (Feringa et al., 1976; Kiernan, 1979). While in 1979 Kiernan had soundly rejected the hypothesis that functional regeneration in the mammalian CNS was impossible, he also argued that the most promising path forward for clinical research was to determine “why, under ordinary conditions, this regenerative process is unsuccessful” (Kiernan, 1979).

Responding to the “misleading claims” that had resulted from prior CNS regeneration studies and citing “wasteful duplication of scientific efforts” as well as “disappointing the paraplegic community,” a 1980 editorial in *Experimental Neurology* even had laid out several “Criteria for Evaluating Spinal Cord Regeneration Experiments” (Guth et al., 1980). Adopted by the National Institute of Neurological and Communicative Disorders and Stroke, these criteria had insisted that any new publications claiming the production of CNS regeneration under experimental conditions verify both loss of function by way of specific structural injury (such as spinal cord transection) and gain of function (such as behavioral recovery) by way of specific structural regrowth. To this day, the lamprey stands out as one of the only vertebrate models for which all these criteria can be upheld.

**FIGURE 7:**
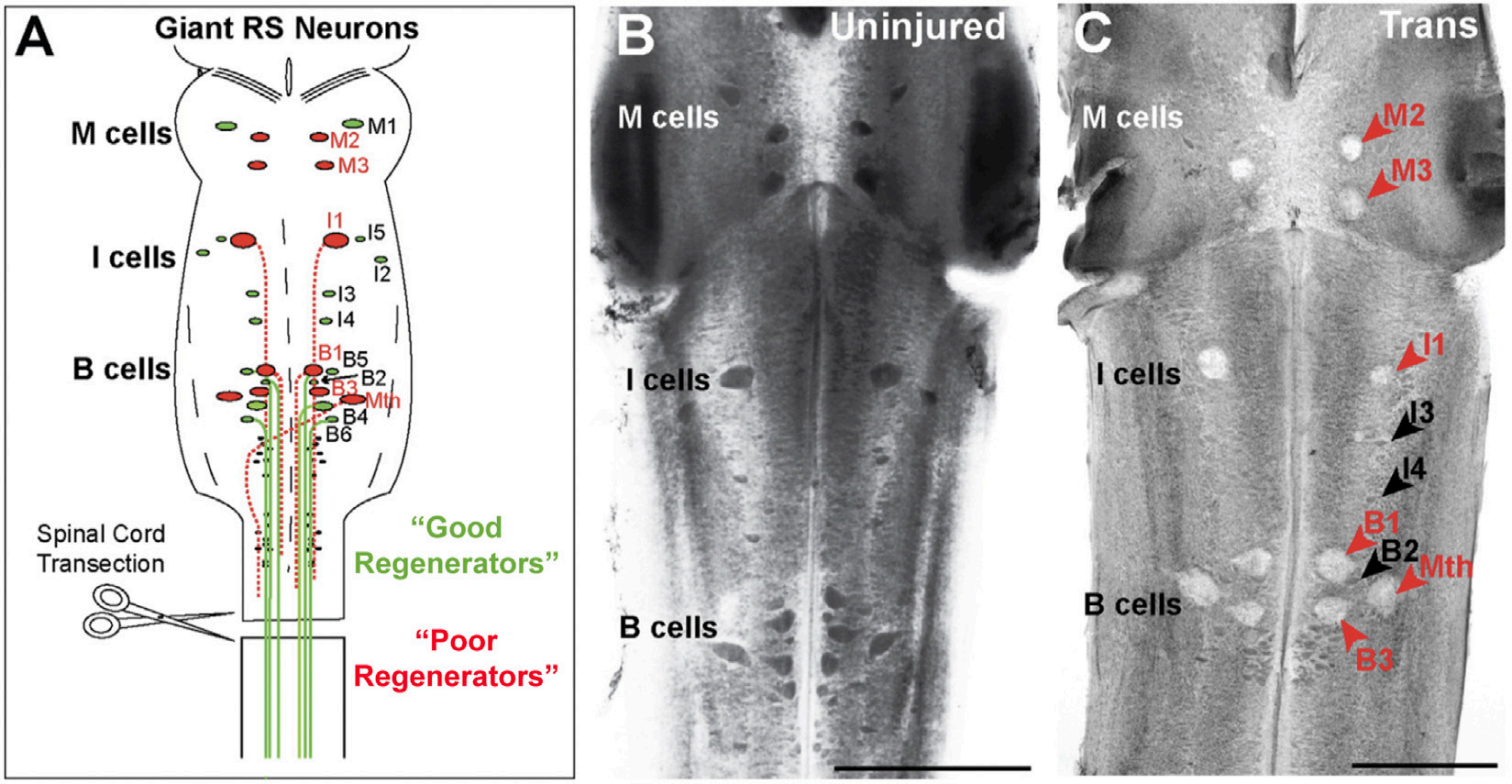
Lamprey giant reticulospinal (RS) neurons have different regeneration capacities. **(A)** Diagram of the lamprey midbrain and hindbrain showing the 30 giant RS neurons. RS neurons are further designated as mesencephalic (M), isthmic (I), bulbar (B), and Mauthner (*M*th) cells. Upon spinal transection, all the giant RS neurons are axotomized, after which a reproducible subset regenerates their axons with high probability (“good regenerators”) while the remaining undergo delayed apoptosis (“poor regenerators”). While most giant RS neurons reside in the midbrain and hindbrain, M1 and M2 are located in the caudal diencephalon (Pombal and Puelles, 1999); moreover, I3 and I4 reside outside of the isthmic region in rhombomeres 2 and 3 of the hindbrain (Murakami et al., 2004). **(B)** Image of a toluidine blue stained uninjured, control lamprey brain showing all 30 giant RS neurons, which are darkly Nissl stained, indicating healthy cells. **(C)** After 11 weeks post-transection (Trans), the “poor regenerators” (red arrows) swell and lose their Nissl staining due to injury-induced cell death, while the “good regenerators” (black arrows) remain healthy. Scale bars = 500 mm. Adapted from Fogerson et al., 2016, and used with permission by Elsevier.

As the molecular era dawned, the lamprey was once again poised to offer new insights in this space. As in previous decades, this was thanks to the multiple scales of analysis made possible by the lamprey’s giant reticulospinal neurons and the broad scope of relevance conferred by the animal’s early evolutionary position. Indeed, the 1990s and 2000s shepherded in the first molecular insights into regeneration. The Human Genome Project, which began in 1990 and concluded in 2004, provided financial support for genome sequencing in the human and several model organisms, including for two animals already popular in neuroscience research: the laboratory mouse, *Mus musculus*, and the nematode, *Caenorhabditis elegans* (Ankeny, 2001; Maxson Jones et al., 2018). In this context, and even in the absence of a sequenced genome, molecular analyses of the lamprey’s large, identified neurons—that is, of features *intrinsic* to these neurons—helped to show conserved features of those CNS neurons that tended to regenerate their axons and those that did not (Figure 7A).

The first demonstrations took place in the Selzer laboratory in the late 1990s, when the regeneration of RS axons was shown to be highly correlated with changes in the expression of neurofilament-180 (NF-180) (Pijak et al., 1996; Jacobs et al., 1997). After injury due to spinal cord transection, all the giant RS neurons initially downregulated NF-180 expression. However, over time, the “good” regenerators (specifically, those with a high probability of regenerating, amounting to approximately half of the pre-injury population) recovered NF-180 expression along a time course resembling anatomical regrowth across the lesion site and functional recovery. In 2008, it also was shown that the “poor” regenerators (the other half of the pre-injury population) undergo delayed cell death, suggesting that protection from apoptosis may promote regeneration (Figures 7B, C) (Shifman et al., 2008). Finally, following publication of the lamprey genome (Smith et al., 2013), Selzer’s group used anti-sense morpholinos to reduce NF-180 expression, demonstrating a functional role for neurofilaments in axon regeneration (Zhang et al., 2015). Such correlation of molecular changes within individual CNS neurons that are “good” and “poor” regenerators, not to mention with axon regeneration and behavioral recovery along a regular time course, remains extremely difficult to accomplish in other experimental animals. In addition, this work lent early credence to the idea that intrinsic factors expressed within neurons could drive or inhibit regeneration, in addition to the extracellular factors, such as glial scarring, that had received so much study since the 1950s, especially in mammals (Guth and Windle, 1970).

In the 1990s and early 2000s, the notion of the significance of intrinsic neuronal factors held little traction outside the lamprey, but it is now widely accepted that intrinsic and extrinsic factors interact closely to regulate regeneration (Ferguson and Son, 2011; Morgan and Shifman, 2014; Morgan, 2017). The classic example in mammals is the differential growth of central and peripheral axons in dorsal root ganglia (Moore et al., 2009; Lerch et al., 2014; Mahar and Cavalli, 2018). Moreover, in lampreys, direct comparisons of neurons with low and high regenerative probabilities have continued to illuminate intrinsic molecular factors that hinder regeneration by causing neurodegeneration, such as the post-injury aggregation of synuclein, a synaptic vesicle-associated protein whose aberrant aggregation is also linked to Parkinson’s and other neurodegenerative diseases (Busch and Morgan, 2012; Fogerson et al., 2016). Studies in the lamprey spinal cord also have provided insights into conserved regulatory pathways that promote axon regeneration in the CNS, such as by way of the second messenger cyclic AMP (cAMP) (Jin et al., 2009; Lau et al., 2013; Pale et al., 2013). Enhancement of axon regeneration by cAMP was first demonstrated in mice, in the laboratory of Marie Filbin in the Department of Biological Sciences of Hunter College at the City University of New York (Qiu et al., 2002; Hannila and Filbin, 2008). This finding has since been corroborated in other vertebrates, such as in the optic nerves of rodents and goldfish, and in invertebrates such as *C. elegans* (Li et al., 2003; Rodger et al., 2005; Ghosh-Roy et al., 2010).

The lamprey model also has helped to corroborate and extend other molecular pathways that influence vertebrate CNS regeneration, including by way of axon guidance molecules, Wnt signaling, ion channels, neurotransmitter systems, and extracellular matrix components (Shifman and Selzer, 2007; McClellan et al., 2008; Shifman et al., 2009; Herman et al., 2018; Romaus-Sanjurjo et al., 2018; Hu et al., 2021). These molecular studies have been conducted primarily on larval sea lampreys in the laboratories of Michael Selzer, Antón Barreiro-Iglesias and María Rodicio (Universidad de Santiago de Compostela, Spain), Jennifer Morgan (The University of Texas at Austin, now the Marine Biological Laboratory in Woods Hole), and their collaborators. Indeed, whole tissue transcriptome analyses have revealed that many of the growth-promoting pathways occurring during mammalian PNS regeneration also are upregulated in the lamprey CNS after spinal cord injuries, including a canonical set of transcription factors identified as “regeneration-associated genes” like Jun, ATF3, Sox11, and several SMADs (Chandran et al., 2016; Herman et al., 2018; Katz et al., 2022). An unbiased transcriptome analysis also has revealed that gene expression changes occur throughout the time course of functional recovery both in the spinal cord *and in the brain*, highlighting the dynamic nature of molecular changes during regeneration and the importance of pro-regenerative responses in supraspinal locations (Herman et al., 2018).

Moreover, while these lines of study have highlighted conserved molecular factors that promote or inhibit CNS regeneration, still further research has emphasized physiological mechanisms promoting regeneration in the lamprey spinal cord, building on and refining research begun in earlier decades. In the 1990s and 2000s, Andrew McClellan’s work correlated cellular regrowth with behavioral recovery in new ways. Working with Grillner and independently, in the 1980s McClellan had studied the mechanisms of “fictive swimming” in the *in vitro* lamprey spinal cord (McClellan and Grillner, 1983; McClellan, 1984). Then, at the Department of Physiology and Biophysics at the University of Iowa and later at the Interdisciplinary Neurosciences Program at the University of Missouri, McClellan and his team used kinematic analyses, electromyography recordings, and retrograde neuronal labeling methods to refine the time course of descending axon regeneration and correlate that with the behavioral recovery of swimming. Amongst the key findings from this work were that regeneration of both descending and ascending axons was robust, but incomplete and variable, over the time course of recovery (McClellan, 1990, 1994; Davis and McClellan, 1993, 1994; Armstrong et al., 2003); that axons continued to regenerate long distances even after behavioral recovery was complete (Davis and McClellan, 1994; Rouse and McClellan, 1997) and that a conditioning lesion also enhances axon regeneration in the lamprey, as occurs in mammals (Zhang et al., 2004). Together, these studies contributed to the idea that the robustness of swimming recovery in the lamprey following spinal cord injury is accompanied by imperfect structural regeneration of axons, setting the stage for understanding synaptic mechanisms that may contribute to this apparent paradox.

Since 2009, David Parker’s work at the University of Cambridge also has built on earlier physiological research, namely, those studies of compensatory plasticity first pioneered in the 1970s. This work has helped to contextualize the molecular studies of intrinsic factors that have unfolded over the last two decades. Using primarily intracellular recordings, Parker’s group has elegantly shown that spinal cord injury changes the intrinsic excitability and synaptic properties of many intraspinal neurons both above and below a lesion site, including in motor neurons, multiple classes of interneurons, and sensory neurons (Cooke and Parker, 2009; Hoffman and Parker, 2011; Becker and Parker, 2019). They also have shown that modulation by 5-HT and other neurotransmitters assists in functional recovery of the spinal central pattern generators (Svensson et al., 2013; Becker and Parker, 2014, 2019). Moreover, Parker’s group recently has corroborated the 1980s finding that regenerated RS synapses can produce postsynaptic responses of normal or enhanced amplitude (Mackler and Selzer, 1987; Parker, 2022). Surprisingly, these robust synaptic responses can occur even though regenerated RS synapses are sparse and have smaller synaptic vesicle clusters than normal, as determined in the Morgan laboratory by electron microscopic analysis, though they seem to retain the proper presynaptic organization (Oliphint et al., 2010). Collectively, these anatomical, physiological, and molecular findings suggest that the regenerated spinal cord is a “new cord”: one with a distributed and varied range of compensatory changes that together re-establish a functional spinal locomotor network, as Parker conveyed in the title of an article in 2017 (Parker, 2017).

Effective therapies for traumatic CNS injuries or diseases, in other words, may require the promotion of certain network- or systems-level properties reflecting entirely new connections. These new connections may include specific patterns of excitation and inhibition within neuronal networks. Additionally, any therapies must consider intricate interactions between intrinsic factors such as gene expression, and extracellular factors, such as the contributions of glial cells.

## 7 Conclusion: The history and future of CNS regeneration research in lampreys

In this article, we have examined the history of one experimental organism, the lamprey, in neurobiology since the 1830s. More specifically, we have argued that large nerve cells in the lamprey’s CNS, in conjunction with the animal’s basal evolutionary position, facilitated studies in spinal cord regeneration research after 1959. Examined with prevailing and new laboratory technologies, the lamprey’s RS neurons enabled fresh insights into conserved attributes of how axon regrowth, compensatory plasticity, and intrinsic molecular factors contribute to functional recovery. But investigators also have long been able to attach a broad scope of relevance to this work, interpreting them as suggesting conserved features of successful, and sometimes even unsuccessful, CNS regeneration. Because lamprey CNS regeneration has offered insights into why basal vertebrates accomplish this feat so well, whereas mammals fare so poorly, such work has persisted in biological and medical institutions, despite only ever encompassing a small proportion of studies in the field overall.

Yet, we believe that examination of historical research also can suggest paths forward, for instance by demonstrating fuller expressions of possible experimental and theoretical approaches than might be exhibited in current research, or by revealing blind spots in present-day intellectual and experimental trajectories. This history has shown how both biological and medical value have been gleaned from a single, non-traditional model organism for which molecular genetics tools only have been developed relatively recently. Despite a decreasing number of lamprey studies in the neurosciences over the last two decades (Figures 6A, B), it also suggests ways in which lampreys could continue to contribute productively to the field.

Since the 1980s, for example, much of the experimental animal research on CNS regeneration has focused on genetically standardized “model” organisms, especially rodents. The defining attributes of these organisms enable biologists to control and evaluate the effects of genetic manipulations and infer the relevance to humans by way of molecular sequence conservation (Strasser, 2008, 2009; Ankeny and Leonelli, 2011, 2021). The power and value of these methods are undeniable. Yet, despite billions spent on regenerative medicine research, the global burdens of CNS injuries and degenerative diseases remain immense (Badhiwala et al., 2019; Hale et al., 2019) and the treatment options limited, although significant improvements have been made (i.e., Young, 2014; Angeli et al., 2018; Courtine and Sofroniew, 2019; Kathe et al., 2022). Developing new ways of moving from specific experiments to general, and perhaps medically relevant, conclusions could be very valuable, and perhaps even exemplified by non-traditional model species such as the lamprey (i.e., Green et al., 2018; Maxson Jones, 2020). Indeed, history of biology tells us that diverse avenues towards producing biological and medical knowledge can co-exist. The conjunction of experimental tractability, by way of large neurons, and evolutionary position that has long perpetuated studies of lampreys can continue to offer biological and clinical insights.

For instance: What intrinsic factors are most important for driving neural regeneration in the CNS? Are there master regulators, like transcription factors, which if upregulated will control whole growth programs? The lamprey can be used to study the cell biology of these processes at the level of individual neurons, enabling side-by-side analyses of those that do and do not regenerate. In addition, this organism provides a platform for identifying conserved molecular pathways that promote regeneration in the vertebrate CNS. Also, how do regenerative processes coordinate across scales (MacCord and Maienschein, 2019, 2021, 2022)? With the new genome and transcriptome resources now available for the lamprey (Smith et al., 2013, 2018; Herman et al., 2018; Timoshevskaya et al., 2023), it is possible to study spinal cord regeneration from the molecular and cellular levels to synaptic mechanisms, circuit physiology, and behavior with more precision than ever before. A recent neuromechanical model of spinal injured lampreys revealed that sensory feedback amplification can enhance functional recovery, opening novel avenues to explore *in situ* while also expanding the lamprey toolkit to include new computational modeling resources (Hamlet et al., 2023). Few experimental organisms offer this possibility, although zebrafish and *C. elegans* models are being developed and deployed more integratively in this space, providing additional opportunities for comparative approaches (Haspel et al., 2021). There are some limitations with the lamprey spinal cord injury model, namely, the lack of standard transgenic approaches for late larval animals due to their advanced age (5–7 years old) and long lifecycle. However, CRISPR-mediated gene editing is now possible in embryos and early larvae (<1 month old) (Square et al., 2015, 2020; Suzuki et al., 2021). Other types of molecular manipulations using morpholinos and pharmacological approaches are also feasible (Zhang et al., 2015; Fogerson et al., 2016; Romaus-Sanjurjo et al., 2018; Rodemer et al., 2020).

Finally, an over-arching conclusion is that a full understanding of CNS regeneration, which spans from the subcellular to the behavioral levels and is prevalent across taxa, requires analyses at the systems level: of features only apparent when the CNS is examined as a coordinated whole composed of interacting parts, including across species to glean evolutionary trends (MacCord and Maienschein, 2019, 2021, 2022). “Our current knowledge should allow us to improve the lives of patients suffering from spinal cord injury,” neurobiologist Andy Fong and his co-authors wrote in 2009, but “consumed with individual pieces of the puzzle,” such as genetic components in relative isolation, “we have failed as a community to grasp the magnitude of the sum of our findings” (Fong et al., 2009; see also, Parker, 2017). Because lampreys can broaden the scope and scale of spinal cord regeneration research, they are poised to provide further novel insights into biology and therapies.

## Data Availability

The original contributions presented in the study are included in the article/Supplementary Material. Further inquiries can be directed to the corresponding author.

## Footnotes

1 “Lamprey,” 2022, *New World Encyclopedia,* https://www.newworldencyclopedia.org/p/index.php?title=Lamprey&oldid=1083390. Accessed 11 April 2023.

2 Chillag, Ian, “A Parasite Pie Fit For A Queen’s Diamond Jubilee,” 2012, NPR, https:// www.npr.org/sections/waitwait/2012/06/03/154196783/a-parasite-fit-for-a-queen. Accessed 11 April 2023.

3 “Sea Lamprey: A Great Lakes Invader,” Great Lakes Fisheries Commission, http://www.glfc.org/sea-lamprey.php. Accessed 11 April 2023.

4 A typed version of Freud’s 1877 manuscript in German (Freud, 1877) was provided from a scan of the original by Lori Straus Communications (https://loristrauscommunications.com/). A rough translation of the manuscript was then generated by Google translate to confirm the main points noted above, which were corroborated by Shepherd in his analyses of Freud’s 1877, 1878, 1882, and subsequent papers (Shepherd, 2016).

5 Purdy, Michael C., “Obituary: Carl Rovainen, professor emeritus, 73,” 2013, *The Source*, https://source.wustl.edu/2013/03/obituary-carl-rovainen-professor-emeritus-73/. Accessed 27 April 2023.
